# Social–Ecological Determinants of Suicidal Ideation Among Sexual and Gender Minority Adults: A Cross-Sectional Study in the United States

**DOI:** 10.3390/healthcare12242540

**Published:** 2024-12-16

**Authors:** Jennifer R. Pharr, Kavita Batra

**Affiliations:** 1 Department of Environmental and Occupational Health, School of Public Health, University of Nevada, Las Vegas, NV 89119, USA; 2 Department of Medical Education, Kirk Kerkorian School of Medicine at UNLV, University of Nevada, Las Vegas, NV 89102, USA; 3 Office of Research, Kirk Kerkorian School of Medicine at UNLV, University of Nevada, Las Vegas, NV 89102, USA

**Keywords:** suicidal ideation, sexual and gender minorities, social-ecological model

## Abstract

**Background/Objectives**: Sexual and gender minority (SGM) adults are at an increased risk for suicide and suicidal ideation compared with cisgender/heterosexual adults. Due to the complexity of suicidal ideation, individual, social, and systemic factors must be considered. The purpose of this study was to identify determinants of suicidal ideation among SGM adults using constructs from the Social–Ecological Model (SEM). **Methods**: This was a cross-sectional study of data collected from 1034 SGM adults between 27 January and 7 February 2022, and variables reflected the four constructs of the SEM, including individual, family, community, and societal levels. Hierarchical multiple linear regression was used to enter the variables in blocks. **Results**: The final model explained 55% of the variance in suicidal ideation, and determinants of suicidal ideation were identified at all four levels. At the individual level, factors were depression (*p* < 0.001, β = 0.231), anxiety (*p* < 0.001, β = 0.279), vigilance distress (*p* = 0.036, β = 0.157), not being out/open (*p* = 0.046, β = 1.136), having some college education (*p* = 0.002, β = −1.875), and personal strength (*p* = 0.012, β = −0.131). Family of origin discrimination distress was significant at the family level (*p* = 0.016, β = 0.174). Social resources (*p* < 0.001, β = −0.113), victimization distress (*p* < 0.001, β = 0.795), and harassment/discrimination distress (*p* = 0.035, β = 0.179) were significant at the community level. At the social level, SGM protective state law score (*p* = 0.43, β = −0.35) and having a proposed or enacted transgender sports ban (*p* = 0.027, β = 1.480) were significant. **Conclusions**: Understanding the factors across the various levels of the SEM that influence suicidal ideation allows for the development of multi-level, social-ecological suicide prevention programs. Such programs can address the unique needs of SGM individuals and help mitigate suicidal ideation.

## 1. Introduction

Sexual and gender minority (SGM) adults are at increased risk for physical [1,2] and mental [1,2,3] health disparities compared with their cisgender/heterosexual peers. Of particular concern is the increased risk for suicide, suicide attempts, and suicidal ideation [4]. Sexual minority adults are 2.7 to 3.8 times more likely to report suicidal ideation and 5.5 to 6.8 times more likely to have attempted suicide in the past year compared with heterosexual adults [4]. Nearly 82% and 39% of gender minority adults reported suicidal thoughts or a suicide attempt in their lifetime, respectively, Kachen et al. [5] compared with 13.5% and 4.6% for the general population, respectively [6]. Given the disparate rates of suicide and suicidal ideation within the SGM community, it is crucial to understand the determinants of suicidal ideation to develop effective programs to reduce risks and enhance protections against suicidal ideation.

While there is no universally accepted definition of suicidal ideation, it generally refers to suicidal thoughts or ideas about suicide, ranging in intensity from fleeting considerations to more serious preoccupations with death [7]. For the purpose of this study, suicidal ideation was defined as the presence of suicidal thoughts and the intensity of those thoughts [8]. Fortunately, the majority of people who have suicidal ideation do not attempt suicide [7]. However, suicidal ideation is one of the strongest predictors of a completed suicide [9]. Addressing determinants of suicidal ideation can reduce risks for suicide. Because of the complexity of suicidal ideation, it is important to employ behavioral health theories and models that go beyond individual characteristics and include the social context when researching this phenomenon [10].

**Figure 1:**
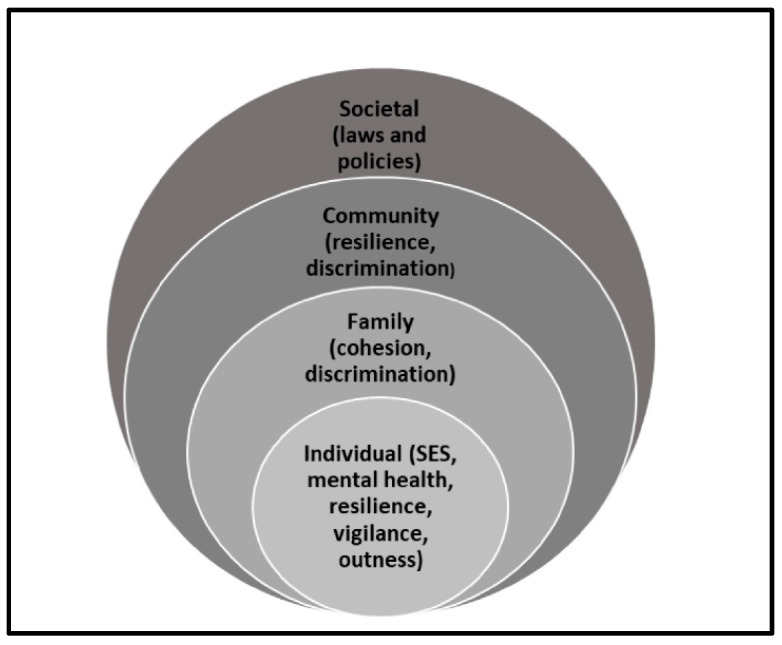
Modified socioecological model of predictors of suicidal ideation among sexual and gender minorities (SES = socioeconomic status).

The Social–Ecological Model (SEM) has been used to understand determinants of health behaviors that exist within the person and within the person’s environment [11,12]. Introduced in the 1970s and formalized as a theory in the 1980s by Urie Bronfenbrenner, the SEM consists of nested levels of influence, with the person at the center surrounded by various social and societal factors [11,12]. Figure 1 illustrates the modified SEM utilized for this study.

At the center are individual characteristics associated with suicidal ideation among SGM people, including sociodemographic characteristics [13,14], mental health (e.g., anxiety, depression, stress (as measured using validated tools explained in the Measures section) [15], personal resilience [16,17,18,19], outness [20], and vigilance [21]. Outness refers to the extent to which an individual discloses their sexual orientation or gender identity in various contexts, whereas vigilance pertains to the constant awareness and anticipation of potential discrimination or stigma [20,21]. One study found that SGM people who identified as female or transgender or were at risk for clinical depression were more likely to report that they had seriously considered committing suicide [14]. Other studies have found that older SGM people [22] or those who have high levels of personal resilience are less likely to report suicidal ideation [23]. SGM people who reported high levels of vigilance (i.e., were guarded so that others do not know their sexual orientation or gender identity) had higher levels of suicidal ideation [24]. Findings regarding the association between outness and suicidal ideation are mixed, with some reporting an increase in suicidal ideation with greater outness [22,25] and others finding that outness was protective against suicidal ideation [20,26].

The second level of influence examines the role of the family in shaping suicidal ideation among sexual and gender minorities (SGMs). Families serve as a critical support system, but their influence can be either protective or detrimental [15,27,28]. Experiences of family discrimination, such as rejection or lack of acceptance due to one’s sexual orientation or gender identity, have been shown to significantly increase the risk of suicidal ideation [15]. In contrast, family cohesion, which is characterized by emotional support, open communication, and acceptance, acts as a protective factor, decreasing the likelihood of suicidal ideation [5,27,28,29,30,31]. These findings highlight the central role of familial dynamics in shaping mental health outcomes for SGM individuals [16,17,18,19,21].

The third level shifts the focus to the broader societal context, encompassing community and societal interactions that influence suicidal ideation. At this level, research consistently shows that experiences of societal discrimination, such as prejudice, stigma, and systemic inequities, are positively associated with suicidal ideation among SGM individuals [5,29,30,31]. Discrimination in social, institutional, or workplace settings can exacerbate feelings of isolation and hopelessness, contributing to poorer mental health outcomes. Conversely, the presence of social resources, including access to supportive networks, affirming community spaces, and inclusive policies, is negatively associated with suicidal ideation [16,17,18,19,21]. These social resources provide a buffer against the negative effects of discrimination by fostering a sense of belonging, validation, and safety within the community. This level underscores the importance of addressing societal factors, such as stigma and marginalization, while promoting inclusive and supportive environments to reduce the risk of suicidal ideation in SGM populations.

The final level of influence encompasses systemic societal factors, including state-level laws and policies that shape the broader environment in which sexual and gender minorities (SGMs) live. Research has consistently demonstrated that laws and policies which explicitly protect SGM individuals from discrimination, such as those ensuring workplace protections, housing rights, and access to healthcare, are negatively associated with suicidal ideation. For example, protective policies have been linked to significantly lower rates of suicidal ideation among SGM individuals by fostering a sense of safety, acceptance, and inclusion [32]. These findings highlight the critical role of systemic change in reducing mental health disparities and underscore the importance of advocating for legal and policy reforms to create a supportive societal framework for SGM populations. The Movement Action Project (MAP)—Equality Map State Profile scores as a measure of state level SGM protective laws and policies. MAP tracks over 50 different SGM-related laws and policies [33,34]. A state’s policy score is reflective of the number of protective laws and policies within each state that shape SGM people’s lives, experiences, and equality. Score range from −6 to 39, with higher scores indicating more SGM-protective laws and policies within a state. Additionally, because MAP tracks laws and policies that have been enacted, it is a more historical reflection of state-level laws and policies.

Conversely, discriminatory laws and policies that marginalize SGM individuals, such as those restricting marriage equalities, adoption rights, or access to gender-affirming care, are positively associated with higher rates of suicidal ideation [21,35,36,37,38,39,40]. These policies reinforce stigma, limit access to essential resources, and perpetuate feelings of exclusion and vulnerability among SGM populations. At the time of data collection, over 300 anti-SGM bills had been introduced across 36 states, with many targeting transgender individuals. From 2020 to February 2022, 10 states enacted transgender sports bans, with 27 others proposing similar restrictions [33,34]. This rise in transgender sports bans had garnered an increased media attention, potentially affected participants’ mental health. Because of this, we included whether a state had proposed or enacted a transgender sports ban as a measure of a discriminatory law/policy.

Most research on suicidal ideation has focused on individual characteristics (biological, psychological, demographic), which is necessary but insufficient [10]. Given the complexity of suicidal ideation, social and systemic factors must be considered in addition to individual characteristics. Utilizing constructs of the SEM provides researchers with the tools to address this complexity by exploring the individual, social, and systemic factors impacting suicidal ideation simultaneously [10]. Factors that were selected at each level of the SEM in this study were those that have been found to influence suicidal ideation among SGM people, either directly or indirectly. However, in this study, multiple factors associated with each level of the SEM were included to identify population-specific risk and protective factors influence suicidal ideation, which has not been done previously.

The purpose of this study was to identify determinants of suicidal ideation among SGM adults using variables within each level of the SEM. The hypotheses were that the addition of different levels of the SEM to the model would influence which factors were significantly associated with suicidal ideation and that more of the variance in suicidal ideation would be explained with each subsequence model.

## 2. Methods

### 2.1. Study Design, Sample, and Recruitment

This was a cross-sectional study of data collected between 28 January and 7 February 2022 from 1033 adults who identified as SGM from across 50 U.S. states and Washington, DC. A contractual agreement was set up with Qualtrics Research Marketing Team to provide a high-quality dataset of the desired sample following a pay-for-services model. Part of the contract was that Qualtrics authenticated unique participants. As part of their model, Qualtrics recruits a panel of participants through convenience sampling of sources acquired from partnerships with over 20 online sample providers. This recruitment method enables Qualtrics to provide the researcher with diverse datasets that represent the population under study. The respondents are first randomly selected by the sample partners through traditional, actively managed, double-opt-in market panels that allow selecting participants who are likely to qualify. Financial incentives paid to the participants were given in variety of forms, including gift cards, cash rewards, SkyMiles, or redeemable points per the contract between Qualtrics and panel providers or data collection partners. Variability in the amount of incentive (which was guided by the contractual terms between Qualtrics and their data collection partners or panel providers) was not revealed to the researcher, nor were the recruitment ads.

Only individuals who identified as SGM, were U.S. residents, were 18+ years old, and agreed to participate were eligible. The initial question on the survey was a screening question (Do you consider yourself to be part of the LGBTQ+ community?). The survey was programmed to terminate automatically for those who answered no. This study was deemed exempt by the lead investigator’s Institutional Review Board (Protocol ID: UNLV-2021-128, 5 November 2021) because no identifiable information was collected. Informed consent was obtained electronically from all subjects involved in the study prior to starting the survey. If participants declined to participate, the survey was programmed to terminate automatically. The survey took 20 min or less to complete.

### 2.2. Quantitative Variables

A detailed description of the variables, tools utilized, and coding or recoding of variables is presented in Table 1. Variables were identified that reflect with the constructs of the SEM, as shown in Figure 1, and their association with suicidal ideation. The validated tools used in this survey included the Suicidal Ideation Scale [8,38], the Center for Epidemiological Studies Depression-Short Form [39], the General Anxiety Disorder-7 Questionnaire [40], the Perceived Stress Scale [41], the Resilience Scale for Adults (RSA) [42], and the Daily Heterosexist Experiences Questionnaire (DHEQ) [43]. The 10-item Suicidal Ideation Scale (SIS) questionnaire was used to assess the presence of suicidal thinking and its intensity. Participants were asked to respond to questions using a five-point Likert scale (1 = never to 5 = always). The SIS has demonstrated high internal consistency (Cronbach’s alpha = 0.91), construct validity for self-harm (r = 0.83, *p* < 0.001), and item–total correlations (rs = 0.45–0.74) [8,38]. The CES-D-10 is a 10-item questionnaire used to measure depression symptoms in research and clinical practice. Internal consistency was reported for pre- and postelection studies, respectively (α = 0.86; α = 0.86) [39]. The GAD-7 is a seven-item questionnaire used to assess the general presence of anxiety symptoms. The internal consistency (pre- and postelection) of the measure was α = 0.94, α = 0.93, respectively [40]. The PSS-10 is a 10-item scale measures the stressfulness of situations in the past month of the participants’ lives. The coefficient alpha for reliability has been found to be 0.84–0.86 for the PSS-10, with substantial reliability [41]. The Resilience Scale for Adults (RSA) measures resilience across six subscales. Item responses range from 1 to 7, with higher scores indicate higher levels of resilience Cronbach’s α for subscale ranges from 0.67 to 0.81 and 0.88 for the total score. The test–retest Pearson correlation ranges from 0.73 to 0.80 for subsections and 0.84 for the total score [42]. The Daily Heterosexist Experiences Questionnaire (DHEQ) questionnaire was used to measure discrimination distress across eight subscales. Scores for distress ranged from 0 (did not happen/not applicable to me) to 5 (bothered me extremely). The DHEQ has acceptable internal consistency (Cronbach’s alpha = 0.92), with good internal reliability for each subscale [43].

Individual-level variables included sociodemographic characteristics, mental health (depression [39], anxiety [40], stress [41]), four characteristics of individual resilience (perception of the self, planned future, structured style, social competence) [42], vigilance discrimination distress [43], outness/openness [44], and comfortability with being SGM. Vigilance discrimination distress was considered an individual-level variable because the questions in the subscale were about identity concealment (e.g., pretending that you have an opposite-sex partner, pretending that you are heterosexual, hiding your relationship from other people) [43]. The Social Competence dimension of the Resilience Scale for Adults (RSA) was also added as an individual factor, which assesses an individual’s ability to interact effectively and adaptively with others in various social contexts. Key items within this dimension include enjoying social interactions, establishing new friendships with ease, and effectively communicating with others. Additionally, social competence emphasizes the ability to find humor in challenging situations and to remain flexible and adaptable in social scenarios. These traits reflect a person’s capacity to build and maintain positive relationships, manage social dynamics, and leverage interpersonal connections as a resource during adversity [42].

Family-level variables were family cohesion [42], family of origin discrimination distress [43], and parenting discrimination distress (e.g., having one’s children reject them due to their sexual orientation or gender identity) [43]. Community-level factors were community support [42] and distress associated with harassment, gender identity discrimination, victimization, vicarious discrimination, isolation, and social resources [43]. Isolation discrimination was considered at the community level because the question for this subscale focused on connection with others (e.g., having difficulty finding SGM friends, finding a partner due to SGM identity). The Social Resources dimension of the Resilience Scale for Adults (RSA) evaluates the extent and quality of an individual’s external support systems and connections. Key items in this dimension focus on having close friends who care, receiving support from others during difficult times, and being able to rely on a network of people outside the immediate family. It also emphasizes a sense of belonging and connection to the community. This dimension underscores the role of social relationships in providing emotional support, practical assistance, and a buffer against stress [42].

Objective measures were used for societal level factors. States’ overall equity numbers calculated by the Movement Advancement Project were used as a measure of historical laws and policies protecting SGM people in the state [45]. The Movement Advancement Project tracks over 50 SGM-related laws and policies and scores them positive for protective laws and policies or negative for harmful laws and policies. Scores range from −10.5 (Alabama) to 43 (Colorado) [46]. Additionally, whether a state has proposed or enacted a transgender sport ban (TGSB) between 2020 and the time of the data collection was used as a more recent snapshot of societal attitudes toward the SGM community [47]. This variable was used because 37 states had proposed or enacted a TGSB, which was exceeded other anti-SGM laws and policies at the time.

### 2.3. Statistical Analysis

**Figure 2:**
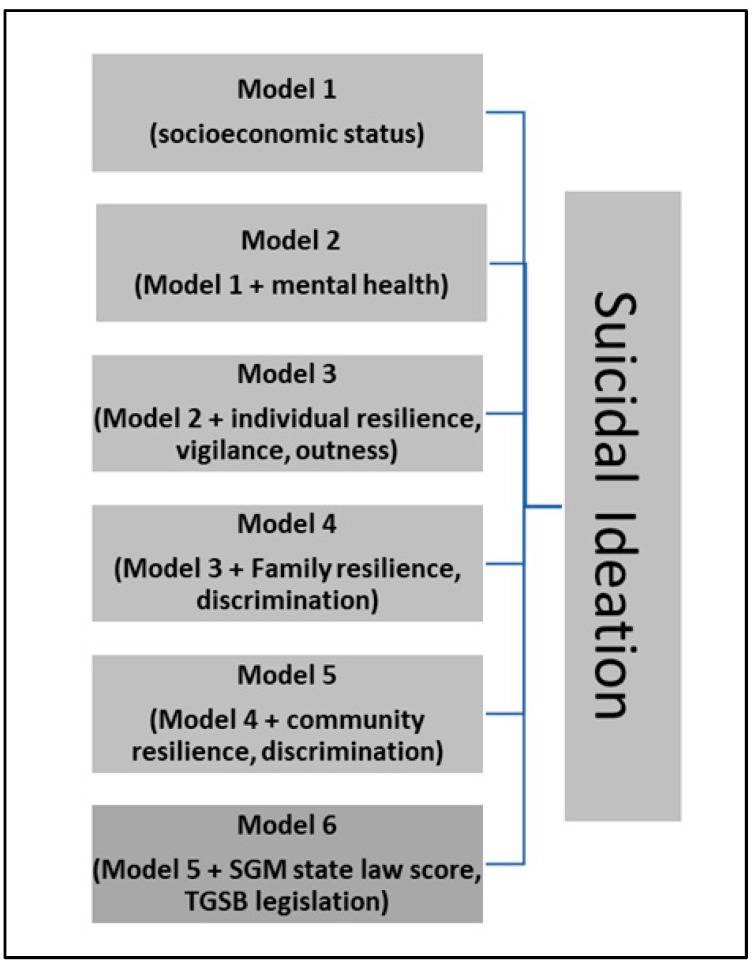
Hierarchical regressions models predicting suicidal ideation among sexual and gender minorities (SGM = sexual and gender minorities; TGSB = transgender sports bans).

Descriptive statistics were conducted for the sample and include mean and standard deviation (SD) for continuous variables and number and percentage for categorical variables. Hierarchical multiple regression was used to enter variables into the analysis in blocks (Figure 2). A total of six models were generated. The first three models included variables at the individual level of the SEM: model 1—sociodemographic variables, model 2—model 1 variables + mental health variables, and model 3—model 2 variables + individual resilience measures, outness, and vigilance. The fourth model added the family level of the SEM: model 3 + family discrimination and cohesion. The fifth model added the community level of the SEM: model 4 + community discrimination and social resources. The final (sixth) model added the social level of the SEM: model 5 + laws and policies. Because of concerns with multicollinearity, we calculated collinearity statistics for each model, which provided the tolerance and variance inflation factor (VIF). In the final (sixth) model, no variable was considered to have severe multicollinearity (VIF > 10) which would have undermined the reliability of the regression model. Only one variable (gender expression discrimination distress) was greater than 5, at 5.5, which would suggest moderate multicollinearity. Because this variable was only slightly above 5, we kept it in the final model. Additionally, 88 participants were missing the outcome variable; they were excluded from the hierarchical multiple regression analysis. Analyses were performed using SPSS. The significance level was set to 0.05, and all *p* values were two-sided.

## 3. Results

### 3.1. Descriptive Statistics

The mean age of the sample was 38.56 years. A majority of the sample identified as non-Hispanic (85%), White (74%), bisexual (46%), and female (55%). More participants reported being employed (48%), single (46%), and having some college education (35%) or a college degree (34%). The income of participants was evenly distributed across the three categories of $50,000 or more (32%), $20,000 to $49,999 (33%), and less than $20,000 (34%). Sixty-one percent of participants reported being open or out, while 68% lived in a state that had proposed or enacted a TGSB. The mean suicidal ideation score was 18.85 (SD = 10.57). Of note, a score of 15 or higher on the Suicidal Ideation Scale is associated with serious suicidal ideation. Means and SDs for the remaining continuous variables are provided in Table 2.

### 3.2. Correlations

As noted in Table 3, suicidal ideation was moderately and positively correlated with anxiety, depression, stress, and discrimination distress at the personal, family, and community levels (*p* < 0.01). However, there was an inverse correlation of suicidal ideation with variables related to personal, community, and family resilience and having more SGM protective state-level laws and policies (*p* < 0.01).

### 3.3. Hierarchical Multiple Regression

Nine-hundred and forty-five participants were included in the hierarchical multiple regression. All models in the hierarchical multiple regression were significant (*p* < 0.001). The change in F was significant (*p* < 0.001) for models except model 6 (*p* = 0.064). R^2^ increased from 0.118 in model 1 to 0.571 in model 6. The adjusted R^2^ for the final model explained 55% of the variance in suicidal ideation (Table 4). Determinants of suicidal ideation were identified at the individual, family, community, and societal levels in the models. At the individual level, factors that were positively associated with the total suicidal ideation score were depression (*p* < 0.001, β = 0.231), anxiety (*p* < 0.001, β = 0.279), vigilance distress (*p* = 0.036, β = 0.157), and not being out/open (*p* = 0.046, β = 1.136). Factors that were negatively associated with the suicidal ideation score at the individual level included having some college education compared with no college education (*p* = 0.002, β = −1.875) and perception of self (*p* = 0.012, β = −0.131).

At the family level, only family of origin discrimination distress was significant and increased the overall suicidal ideation score (*p* = 0.016, β = 0.174). At the community level, social resources (*p* < 0.001, β = −0.113) were negatively associated with the suicidal ideation score, while victimization discrimination distress (*p* < 0.001, β = 0.795) and harassment/discrimination distress (*p* = 0.035, β = 0.179) were positively associated with the suicidal ideation score.

Finally, at the social level, the SGM-protective state law score was associated with a decrease in the overall suicidal ideation score (*p* = 0.43, β = −0.35), and having a TGSB proposed or enacted was associated with an increase in the overall suicidal ideation score (*p* = 0.027, β = 1.480).

The collinearity statistics for each model can be found in Appendix A. The 95% confidence intervals for the beta coefficients can be found in Appendix B.

## 4. Discussion

Suicidal ideation is a complex, multifactorial phenomenon. The final model in this study revealed that factors contributing to suicidal ideation exist at each level of the Social–Ecological Model (SEM) when considered simultaneously. In this model, beta coefficients were calculated while controlling for all other variables at each level of the SEM. Statistically significant coefficients indicate that a particular variable has a unique association with suicidal ideation when accounting for other variables, while non-significant coefficients imply that, after accounting for all other variables, the given variable does not have a unique association with suicidal ideation. It is important to note that, for variables that serve as multiple measures of similar constructs (e.g., resilience subscales), achieving significant coefficients can be challenging.

At the individual level, variables were identified that showed both negative and positive associations with suicidal ideation scores. Among the four resilience measures, only self-perception emerged as a protective factor against suicidal ideation. Participants with higher self-perception scores had a stronger belief in their ability to solve problems, trust their judgment, and rely on their capabilities. A study comparing individuals who had attempted suicide with those who had not found that non-attempters scored higher in self-perception [48]. Additionally, individuals with high self-perception scores exhibited lower depression and anxiety levels [49]. Enhancing individual resilience may help reduce suicidal ideation, particularly for individuals with other stress-related psychiatric conditions, such as depression and anxiety [50].

Higher depression and anxiety scores were associated with increased suicidal ideation scores. This finding aligns with previous research showing a positive linear relationship between depression and/or anxiety disorders and both psychological distress and suicidal ideation, evident in the general population [51] and among SGM individuals [52]. A recent review identified depressive disorders and comorbid depression and anxiety disorders as strong predictors of suicidal ideation [51]. SGM individuals with elevated depression and/or anxiety scores should be monitored for suicidal ideation, as treatment may differ for those diagnosed with both depression and suicidal ideation compared to those with depression alone [53].

Beyond depression and anxiety, suicidal ideation scores also increased with distress related to concealing relationships or pretending to be heterosexual (vigilance). Additionally, participants who were not open about their identity exhibited higher suicidal ideation scores. Research on outness remains inconclusive, with some studies finding it protective against suicidal ideation [20,26] and others finding it to be a risk factor [54,55]. However, age appears to play a role in whether outness increases or decreases suicide risk, with some research indicating that disclosing a minority sexual orientation or gender identity may elevate suicide risk for adolescents, who may experience family rejection [25,54]. Conversely, SGM adults who are open about their identities report a decreased risk of suicidal ideation [20,26].

At the family level of the SEM, family origin discrimination distress was linked to higher suicidal ideation. This is consistent with other research finding that family rejection is associated with an increased risk of suicidal ideation among SGM individuals [5,56,57,58]. Previous studies have shown that family rejection corresponds with increased suicide odds as rejection intensity rises [58]. Marzetti and colleagues suggest that family rejection of SGM identities is, in part, a consequence of entrenched cisgender/heteronormative societal norms, which need to be challenged to reduce SGM-related suicidal ideation [59].

At the community level of the SEM, stronger social support was associated with decreased suicidal ideation scores. Participants with higher social support scores were more likely to report having friends or family members with whom they could discuss personal issues and who they felt valued and encouraged by. These types of social resources have been shown to reduce suicidal ideation risk in other studies and underscore the importance of fostering external support to alleviate mental distress [18,19,60,61,62]. This support can be strengthened through various means, such as connecting individuals to SGM-affirming groups [63] or SGM-oriented social media platforms [64], which can provide camaraderie and bolster support among SGM individuals in communities or countries with anti-SGM cultural climates, limited protections, or prevalent biases.

Also at the community level, distress from harassment, discrimination, and victimization was found to heighten suicidal ideation. In the DHEQ, harassment and discrimination questions included experiences such as verbal harassment, derogatory name-calling, or unfair treatment in public spaces due to SGM identity. Victimization questions addressed experiences like physical assault, including being punched, kicked, assaulted with a weapon, or sexually assaulted. Other studies have found similar associations between these forms of discrimination and suicidal ideation [5,52,65,66]. Most research on interventions to reduce harassment, discrimination, or victimization focuses on school environments and intimate partner violence rather than the broader community. As noted, addressing pervasive cisgender/heteronormative climates is necessary, though specific methods for doing so warrant further research.

Countering anti-SGM harassment, discrimination, and victimization through SGM-protective state and federal laws may initiate cultural change around SGM stigma. In this study, participants residing in states with more SGM-protective laws and policies had a lower risk of suicidal ideation, while those in states with policies excluding transgender athletes from sports had a higher risk. This finding aligns with previous research on the relationship between laws and suicidal ideation [33,34]. State-level exclusionary laws may either reflect or shape societal-level discriminatory conditions or cultural norms [67]. Regardless, research consistently indicates that greater protections for SGM individuals and fewer exclusionary laws are linked to improved mental health outcomes [21,33,34,35]. Federal and state legislators should consider the impact of their laws and policies on SGM communities, as these can either bolster SGM protections or contribute to further marginalization.

This study is not without limitations. As a cross-sectional study, it cannot infer causation. Additionally, self-reported survey responses carry the potential for bias, as participants may respond in socially desirable ways rather than truthfully. Self-selection bias may also be a factor, as those interested in this topic may have been more likely to participate. Other unmeasured confounders could further explain the variance in suicidal ideation.

Despite these limitations, this study contributes to understanding suicidal ideation among SGM individuals by examining multiple intrapersonal and external factors across the SEM simultaneously. The study identifies significant factors uniquely associated with suicidal ideation after controlling for all other factors at each SEM level. It is also the first study to evaluate suicidal ideation factors across four levels of the SEM, providing insights for developing targeted suicide prevention programs.

## 5. Conclusions

A comprehensive understanding of the factors influencing suicidal ideation across different levels of the Social–Ecological Model (SEM) provides a framework for developing effective, multi-level Social–Ecological Suicide Prevention Programs (SESPPs) tailored to the needs of sexual and gender minority (SGM) populations. Based on the findings from this study, a successful program should include several key components across various levels of intervention. At the individual level, it is essential to offer SGM-supportive mental health services that promote resilience and to address depression and anxiety, particularly for individuals with suicidal ideation. Programs that foster self-acceptance and enhance coping skills can also play a vital role. At the family level, initiatives like family acceptance programs, such as those implemented by San Francisco State University, can help reduce stigma and promote understanding. The community level should focus on providing safe spaces, SGM-affirming resources, and platforms—such as SGM-tailored social media—that work to increase visibility, acceptance, and normalization of the SGM community. Anti-discrimination messaging is also crucial at this level. Finally, at the societal level, advocating for stronger state and federal laws and policies that protect SGM individuals from discrimination is essential to creating a supportive environment for long-term mental health. To truly gauge the effectiveness of these interventions, further research is needed to assess the impact of multi-level SESPPs on suicidal ideation within SGM populations.

## Figures and Tables

**Table 1:** Variables and measures used in this study.

Variable	Tool Used	Coding
Dependent Variable		
Suicidal Ideation	The 10-item Suicidal Ideation Scale (SIS) questionnaire to assess the presence of suicidal thinking and its intensity.	Scores for each question were summed into a SIS score ranging from 10 to 50, with a higher score representing a greater intensity of suicidal thoughts. Continuous variable.
Independent Variables		
**Individual Factors** *Sociodemographic*	(1) Sexual orientation—(lesbian, gay, bisexual, queer, questioning, asexual, and straight/heterosexual.	(1) Recoded—categories other than lesbian, gay, or bisexual were grouped as ‘other’.
	(2) Gender identity—(female; male; trans man, trans male; trans woman, trans female; genderqueer; gender non-conforming, gender non-binary).	(2) Recoded—categories other than female, male, and transgender/non-binary were grouped as ‘other’.
	(3) Education—less than high school, high school diploma/GED, some college/no degree, Associate’s degree, Bachelor’s degree, Master’s degree, Doctoral degree, or professional.	(3) Recoded—high school degree or less, some college/no degree/Associate’s degree, Bachelor’s or higher.
	(4) Employment—employed, retired, homemaker, student, unable to work, unemployed and looking for work.	(4) Recoded—employed, out of the labor force, unable to work, unemployed.
	(5) Income—less than $10,000, $10,000–19,999, $20,000–29,999, $30,000–49,999, $50,000–74,999, greater than $50,000.	(5) Recoded—less than $20,000, $20,000–49,999, greater than $50,000.
	(6) Marital status—divorced, separated, widowed, married, member of an unmarried couple, single (never married).	(6) Recoded—single (previously married), married/coupled, single (never married).
	(7) Race—American Indian/Alaskan Native, Asian, Black, Native Hawaiian/Pacific Islander, Other, Multiple.	(7) Recoded—Black, White, Other races, Multiple races.
	(8) Ethnicity—Hispanic, Spanish, Latinx or Non-Hispanic.	(8) Dichotomous variable.
	(9) Age	(9) Continuous variable.
*Mental Health*	Depression—The Center for Epidemiological Studies Depression-Short Form (CES-D-10)	Continuous variable on a scale. Higher score equaled greater depression.
	Anxiety—General Anxiety Disorder-7 Questionnaire (GAD-7):	Continuous variable on a scale. Higher score equaled greater anxiety.
	Stress—Perceived Stress Scale (PSS-10)	Continuous variable on a scale. Higher score equaled greater stress.
*Resilience*	Resilience Scale for Adults (RSA) measures resilience across multiple subscales (Perception of the Self, Planned Future, Structured Style, Social Competence) that assess interpersonal measures of resilience.	Higher score for each subscale equals great resilience. Continuous variable.
*Vigilance*	The Daily Heterosexist Experiences Questionnaire (DHEQ)	Subscale of Vigilance distress. Higher score equals greater distress.
*Outness*	Outness—I would say that I am open (out) as LGBTQ+ person. Likert scale of 1 = not at all open/out to 5 = totally open/out.	Dichotomized with not at all to somewhat out/open = not out/open and out/open to totally open/out = out/open.
*Personal comfortability with being SGM*	Three statements on a Likert scale of 1 = strongly disagree to 7 = strongly agree. ‘Even if I could change my sexual orientation and/or gender identity, I wouldn’t.’, ‘I feel comfortable being an LGBTQ+ person’, and ‘Being LGBTQ+ is as natural as being heterosexual/cisgender’.	Scores were summed, with a higher score equaling greater comfortability.
**Family Factors** *Family Discrimination*	The DHEQ questionnaire (see above).	Subscale of Family of origin discrimination distress and Parenting discrimination distress. Higher scores equal greater distress.
*Family cohesion*	Resilience Scale for Adults (see above).	Subscale of Family cohesion. Higher score equals greater cohesion.
**Community Factors** *Social resources*	Resilience Scale for Adults (see above).	Subscale of Social resources. Higher score equals greater social resources.
*Community discrimination distress*	The DHEQ questionnaire (see above).	Subscales of Harassment/discrimination distress, Gender identity discrimination distress, Victimization discrimination distress, Vicarious discrimination distress, Isolation discrimination distress. Higher scores equal greater discrimination distress.
**Societal** *SGM State Law Score*	Movement Action Project—Equality Map State Profile.	Scores were retrieved February 2022 and range from −6 to 39. Higher scores equal more SGM-protective state-level laws and policies.
*Transgender Sports Bans*	ACLU 2021 Session bills.	Dichotomized as yes—proposed or enacted TGSB or no—TGSB not proposed or enacted.

**Table 2:** Characteristics of the sample (N = 945).

Variables	Categories	Statistics
**Individual—Socioeconomic**		
Age (M ± SD)	-	38.56 (15.719)
Ethnicity	Hispanic, Latino, or Spanish—No	878 (85.0)
	Yes	150 (14.5)
	Missing	5 (0.5)
Education	College graduate 4 years+	355 (34.4)
	Some college, Associate’s degree	364 (35.2)
	High school graduate or less	309 (29.9)
	Missing	5 (0.5)
Employment	Employed	497 (48.1)
	Unemployed	107 (10.4)
	Out of labor force	304 (29.4)
	Unable to work	119 (11.5)
	Missing	6 (0.6)
Race	White	768 (74.3)
	Black	129 (12.5)
	Other	81 (7.8)
	Multiple	55 (5.3)
Income	$50,000+	333 (32.2)
	$20,000–49,999	34 (33.5)
	<$20,000	348 (33.7)
	Missing	6 (0.6)
Sexual Orientation	Other, multiple sexual orientations	149 (14.4)
	Lesbian	166 (16.1)
	Bisexual	477 (46.2)
	Gay	232 (22.5)
	Missing	9 (0.9)
Gender Identity	Female	572 (55.4)
	Male	327 (31.7)
	Non-binary	58 (5.6)
	Transgender male	15 (1.5)
	Transgender female	11 (1.1)
	Other	4 (0.4)
	Multiple gender identity	46 (4.5)
Marital Status	Single (never married)	470 (45.5)
	Married or unmarried couple	418 (40.5)
	Divorced, widowed, separated	140 (13.6)
	Missing	5 (0.5)
**Individual—Mental Health**		
Depression (M ± SD)	-	12.38 (6.69)
Stress (M ± SD)	-	19.58 (8.00)
Anxiety (M ± SD)	-	9.30 (6.38)
**Individual—Resilience, Vigilance, Outness, Social Competence**		
Perception of self (M ± SD)	-	26.63 (8.09)
Planned future (M ± SD)	-	17.20 (6.40)
Structured style (M ± SD)	-	17.54 (5.17)
Social competence (M ± SD)	-	24.09 (7.77)
Vigilance discrimination distress (M ± SD)	-	10.73 (5.90)
Personal comfortability with being SGM (M ± SD)	-	17.16 (3.94)
Outness	Out/open—yes	634 (61.4)
	Out/open—no	341 (33.0)
	Missing	58 (5.6)
**Family—Resilience and Discrimination**		
Family cohesion (M ± SD)	-	24.34 (8.20)
Family of origin discrimination distress (M ± SD)	-	10.04 (5.94)
Parenting discrimination distress (M ± SD)	-	1.97 (2.50)
**Community—Resilience and Discrimination**		
Social resources (M ± SD)	-	33.74 (9.37)
Harassment and discrimination distress (M ± SD)	-	10.16 (5.97)
Gender expression discrimination distress (M ± SD)	-	9.72 (5.78)
Victimization discrimination distress (M ± SD)	-	5.72(3.72)
Vicarious discrimination distress (M ± SD)	-	17.62 (7.57)
Isolation discrimination distress (M ± SD)	-	7.50 (4.13)
**Societal—State Laws and Policies**		
SGM state law score	-	18.63 (16.40)
Lives in a state with a proposed or enacted TGSB	-	706 (68.3)
No TGSB proposed or enacted	-	327 (31.7)

M = Mean, SD = standard deviation. All values are presented as frequencies and proportions unless stated otherwise.

**Table 3:** Pearson correlations for study variables in the sample population (N = 945).

Variables	1	2	3	4	5	6	7	8	9	10	11	12	13	14	15	16	17	18	19
1. Suicidal ideation	1																		
2. Depression	0.507 **	1																	
3. Stress	0.454 **	0.788 **	1																
4. Anxiety	0.526 **	0.793 **	0.756 **	1															
5. Vigilance discrimination distress	0.479 **	0.245 **	0.205 **	0.312 **	1														
6. Perception of self	−0.433 **	−0.633 **	−0.675 **	−0.578 **	−0.190 **	1													
7. Planned future	−0.384 **	−0.601 **	−0.623 **	−0.486 **	−0.178 **	0.752 **	1												
8. Structured style	−0.292 **	−0.426 **	−0.431 **	−0.364 **	−0.121 **	0.488 **	0.477 **	1											
9. Social competence	−0.257 **	−0.428 **	−0.408 **	−0.384 **	−0.146 **	0.548 **	0.520 **	0.336 **	1										
10. Family cohesion	−0.256 **	−0.374 **	−0.342 **	−0.318 **	−0.112 **	0.387 **	0.439 **	0.270 **	0.401 **	1									
11. Family of origin discrimination distress	0.496 **	0.195 **	0.162 **	0.273 **	0.696 **	−0.135 **	−0.090 **	−0.053	−0.050	−0.147 **	1								
12. Parenting discrimination distress	0.451 **	0.126 **	0.143 **	0.189 **	0.559 **	−0.148 **	−0.064 *	−0.115 **	−0.068 *	−0.022	0.642 **	1							
13. Social resource	−0.409 **	−0.438 **	−0.402 **	−0.359 **	−0.222 **	0.469 **	0.534 **	0.293 **	0.496 **	0.620 **	−0.218 **	−0.166 **	1						
14. Harassment and discrimination distress	0.476 **	0.197 **	0.173 **	0.280 **	0.743 **	−0.138 **	−0.104 **	−0.099 **	−0.045	−0.067 *	0.776 **	0.681 **	−0.164 **	1					
15. Gender expression discrimination distress	0.542 **	0.271 **	0.227 **	0.322 **	0.762 **	−0.179 **	−0.144 **	−0.138 **	−0.108 **	−0.075 *	0.763 **	0.678 **	−0.192 **	0.815 **	1				
16. Victimization discrimination distress	0.544 **	0.159 **	0.127 **	0.234 **	0.600 **	−0.112 **	−0.075 *	−0.087 **	−0.032	−0.020	0.691 **	0.727 **	−0.172 **	0.739 **	0.751 **	1			
17. Vicarious discrimination distress	0.181 **	0.250 **	0.216 **	0.247 **	0.396 **	−0.125 **	−0.111 **	−0.116 **	−0.077 *	−0.042	0.324 **	0.096 **	0.000	0.410 **	0.361 **	0.184 **	1		
18. Isolation discrimination distress	0.440 **	0.271 **	0.223 **	0.293 **	0.700 **	−0.212 **	−0.213 **	−0.130 **	−0.172 **	−0.133 **	0.599 **	0.474 **	−0.265 **	0.605 **	0.692 **	0.540 **	0.350 **	1	
19. SGM state law score	−0.084 **	−0.094 **	−0.122 **	−0.129 **	−0.047	0.082 *	0.051	0.049	0.040	0.037	−0.028	−0.025	0.046	−0.047	−0.009	−0.023	−0.017	−0.018	1

* *p* < 0.05; ** *p* < 0.01.

**Table 4:** Hierarchical multiple regression predicting suicidal ideation among sexual and gender minority adults (N = 945).

Variables	Model 1		Model 2		Model 3		Model 4		Model 5		Model 6	
	B	*p*-Value	B	*p*-Value	B	*p*-Value	B	*p*-Value	B	*p*-Value	B	*p*-Value
**Constant**	25.590	-	10.798	-	16.709	-	15.794	-	17.193	-	17.603	-
**Individual—Socioeconomic**												
Age	−0.176	<0.001 **	−0.059	0.024 *	−0.017	<0.001 **	−0.008	0.721	−0.016	0.467	−0.017	0.441
Hispanic, Latino, or Spanish (ref. Non-Hispanic)	0.819	0.418	1.325	0.127	0.219	0.467	−0.418	0.579	−0.378	0.603	−0.514	0.480
Some college, Associate’s degree	−2.628	0.002 *	−2.495	<0.001 **	−2.457	0.781	−2.143	<0.001 **	−1.844	0.002 *	−1.875	0.002 *
College graduate 4 years+ (ref. high school grad or less)	−1.541	0.111	0.062	0.940	−0.554	<0.001 **	−0.139	0.848	−0.355	0.614	−0.353	0.615
Unemployed	−0.062	0.959	−1.337	0.203	−0.289	0.467	−0.180	0.843	−0.387	0.659	−0.457	0.602
Out of labor force	−0.347	0.679	0.101	0.888	0.444	0.761	0.789	0.204	0.943	0.116	0.992	0.098
Unable to work (ref. employed)	1.871	0.118	−0.358	0.730	0.613	0.494	0.862	0.340	1.090	0.211	1.030	0.238
White	0.643	0.682	0.963	0.474	−0.105	0.517	0.157	0.892	−0.052	0.963	−0.048	0.966
Black	3.835	0.031 *	4.631	0.002 *	2.917	0.931	2.774	0.036 *	2.074	0.104	2.085	0.102
Other (ref. multiple)	2.456	0.211	3.076	0.068	2.858	0.035 *	2.234	0.124	1.727	0.220	1.775	0.208
$20,000–49,999	−0.679	0.440	−1.025	0.175	−1.048	0.061	−1.104	0.090	−1.052	0.095	−1.061	0.091
$50,000+ (ref. <$20,000)	−0.719	0.473	0.316	0.714	0.082	0.125	0.044	0.953	0.147	0.838	0.203	0.777
Other, multiple sexual orientations	0.965	0.492	0.531	0.660	0.052	0.917	−0.335	0.748	−0.533	0.596	−0.505	0.616
Bisexual	1.161	0.319	−0.523	0.602	−0.352	0.962	−0.878	0.316	−0.979	0.247	−0.928	0.271
Lesbian (ref. gay)	0.702	0.620	0.001	1.000	−0.646	0.700	−1.132	0.279	−0.977	0.333	−0.926	0.358
Female	−2.620	0.016 *	−2.661	0.005 *	−1.104	0.555	−0.666	0.415	−0.088	0.911	−0.174	0.826
Transgender, nonbinary	0.342	0.818	−1.631	0.204	−1.310	0.196	−0.648	0.559	0.082	0.940	0.086	0.937
Other, multiple gender identity (ref. male)	−1.303	0.495	−2.626	0.109	−1.716	0.259	−0.669	0.637	0.271	0.846	0.190	0.892
Married	−0.178	0.868	0.239	0.795	0.428	0.247	0.341	0.668	−0.143	0.853	−0.192	0.803
Divorced, widowed, separated (ref. single)	2.609	0.016 *	1.421	0.125	0.504	0.607	−0.006	0.994	0.070	0.928	0.117	0.880
**Individual—Mental Health**												
Depression	**-**	**-**	0.399	<0.001 **	0.260	<0.001 **	0.273	<0.001 **	0.230	<0.001 **	0.231	<0.001 **
Stress	**-**	**-**	0.068	0.283	0.006	0.927	0.007	0.908	0.016	0.777	0.012	0.832
Anxiety	**-**	**-**	0.498	<0.001 **	0.313	<0.001 **	0.291	<0.001 **	0.280	<0.001 **	0.279	<0.001 **
**Individual—Resilience, Vigilance, and Outness**												
Perception of self	**-**	**-**	**-**	**-**	−0.157	0.006 *	−0.117	0.030 *	−0.127	0.015 *	−0.131	0.012 *
Planned future	**-**	**-**	**-**	**-**	−0.075	0.255	−0.107	0.096	−0.051	0.415	−0.049	0.435
Structured style	**-**	**-**	**-**	**-**	−0.099	0.095	−0.096	0.090	−0.091	0.097	−0.088	0.106
Social competence	**-**	**-**	**-**	**-**	0.033	0.410	0.029	0.461	0.061	0.119	0.060	0.127
Vigilance discrimination distress	**-**	**-**	**-**	**-**	0.596	<0.001 **	0.201	0.002 *	0.162	0.030 *	0.157	0.036 *
Outness (ref. out/open)					3.119	<0.001 **	1.420	0.015 *	1.224	0.032 *	1.136	0.046 *
Personal comfortability with being SGM					−0.240	<0.001 **	−0.115	0.102	−0.102	0.140	−0.091	0.191
**Family—Resilience and Discrimination**												
Family cohesion	**-**	**-**	**-**	**-**	**-**	**-**	−0.052	0.141	0.000	0.994	−0.003	0.947
Family of origin discrimination distress	**-**	**-**	**-**	**-**	**-**	**-**	0.304	<0.001 **	0.176	0.015 *	0.174	0.016 *
Parenting discrimination distress	**-**	**-**	**-**	**-**	**-**	**-**	0.894	<0.001 **	0.317	0.064	0.331	0.053
**Community—Resilience and Discrimination**												
Social resources	**-**	**-**	**-**	**-**	**-**	**-**	**-**	**-**	−0.134	<0.001 **	−0.133	<0.001 **
Harassment and discrimination distress	**-**	**-**	**-**	**-**	**-**	**-**	**-**	**-**	−0.178	0.036 *	−0.179	0.035 *
Gender expression discrimination distress	**-**	**-**	**-**	**-**	**-**	**-**	**-**	**-**	0.126	0.180	0.133	0.156
Victimization discrimination distress	**-**	**-**	**-**	**-**	**-**	**-**	**-**	**-**	0.801	<0.001 **	0.795	<0.001 **
Vicarious discrimination distress	**-**	**-**	**-**	**-**	**-**	**-**	**-**	**-**	−0.019	0.630	−0.021	0.587
Isolation discrimination distress	**-**	**-**	**-**	**-**	**-**	**-**	**-**	**-**	−0.010	0.910	−0.016	0.861
**State—Laws and Policies**												
SGM state law score	**-**	**-**	**-**	**-**	**-**	**-**	**-**	**-**	**-**		−0.039	0.043 *
TGSB proposed or enacted (ref. no)	**-**		**-**	**-**	**-**	**-**	**-**	**-**	**-**	**-**	1.480	0.027 *
R^2^	0.118	-	0.355	-	0.481	-	0.530	-	0.569	-	0.571	-
ΔR^2^	0.118	-	0.237	-	0.126	-	0.049	-	0.039	-	0.003	-
F	6.197	-	22.066	-	28.270	-	31.147	-	30.592	-	29.348	-

* *p*-value < 0.05; ** *p*-value < 0.001. Adjusted R^2^ of suicidality in the final model = 0.552; ref. = reference category; grad = graduate; B = beta coefficient.

## Data Availability

Data will be available from the corresponding authors upon request.

## Appendix A

**Table A1:** Collinearity statistics.

Variables	Model 1		Model 2		Model 3		Model 4		Model 5		Model 6	
**Individual—Socioeconomic**	**Tolerance**	**VIF**	**Tolerance**	**VIF**	**Tolerance**	**VIF**	**Tolerance**	**VIF**	**Tolerance**	**VIF**	**Tolerance**	**VIF**
Age	0.518	1.930	0.480	2.085	0.464	2.156	0.460	2.173	0.456	2.193	0.456	2.195
Hispanic, Latino, or Spanish (ref. non-Hispanic)	0.830	1.205	0.828	1.208	0.815	1.227	0.807	1.239	0.804	1.244	0.798	1.253
Some college, Associate’s degree	0.668	1.496	0.667	1.499	0.661	1.512	0.658	1.520	0.652	1.533	0.652	1.534
College graduate 4 years+ (ref. high school grad or less)	0.506	1.976	0.500	2.000	0.484	2.066	0.482	2.076	0.476	2.103	0.475	2.105
Unemployed	0.800	1.250	0.795	1.257	0.785	1.274	0.783	1.277	0.775	1.291	0.772	1.295
Out of labor force	0.730	1.371	0.729	1.373	0.723	1.384	0.720	1.390	0.715	1.398	0.714	1.401
Unable to work (ref. employed)	0.722	1.385	0.703	1.422	0.688	1.453	0.686	1.458	0.678	1.475	0.674	1.483
White	0.231	4.331	0.231	4.335	0.228	4.380	0.228	4.390	0.226	4.423	0.226	4.423
Black	0.305	3.276	0.304	3.290	0.301	3.323	0.299	3.348	0.297	3.370	0.297	3.371
Other (ref. multiple)	0.395	2.533	0.394	2.537	0.391	2.559	0.390	2.567	0.384	2.604	0.382	2.617
$20,000–49,999	0.621	1.611	0.615	1.627	0.612	1.634	0.609	1.641	0.605	1.652	0.605	1.653
$50,000+ (ref. <$20,000)	0.482	2.077	0.478	2.093	0.476	2.102	0.473	2.113	0.468	2.136	0.467	2.141
Other, multiple sexual orientations	0.461	2.171	0.458	2.183	0.454	2.204	0.453	2.209	0.448	2.235	0.447	2.236
Bisexual	0.316	3.161	0.313	3.199	0.306	3.272	0.304	3.292	0.301	3.321	0.301	3.327
Lesbian (ref. gay)	0.400	2.501	0.399	2.507	0.397	2.517	0.396	2.525	0.394	2.539	0.394	2.541
Female	0.365	2.741	0.363	2.755	0.353	2.829	0.351	2.852	0.344	2.905	0.343	2.913
Transgender, nonbinary	0.630	1.586	0.622	1.609	0.618	1.617	0.614	1.629	0.580	1.724	0.580	1.724
Other, multiple gender identity (ref. male)	0.709	1.411	0.706	1.416	0.697	1.434	0.692	1.444	0.660	1.516	0.658	1.521
Married	0.386	2.591	0.385	2.596	0.381	2.625	0.377	2.651	0.373	2.680	0.371	2.697
Divorced, widowed, separated (ref. single)	0.373	2.683	0.370	2.701	0.367	2.727	0.361	2.772	0.358	2.795	0.356	2.809
**Individual—Mental Health**												
Depression			0.274	3.651	0.256	3.904	0.255	3.923	0.248	4.037	0.247	4.044
Stress			0.309	3.239	0.264	3.786	0.264	3.790	0.263	3.806	0.262	3.819
Anxiety			0.308	3.246	0.289	3.455	0.288	3.473	0.286	3.500	0.285	3.507
**Individual—Resilience, Vigilance, and Outness**												
Perception of self					0.309	3.236	0.307	3.255	0.306	3.263	0.305	3.274
Planned future					0.354	2.821	0.343	2.916	0.331	3.017	0.330	3.031
Structured style					0.686	1.458	0.677	1.477	0.673	1.485	0.673	1.486
Social competence					0.650	1.538	0.621	1.611	0.584	1.713	0.584	1.713
Vigilance discrimination distress					0.807	1.239	0.413	2.421	0.275	3.637	0.274	3.649
Outness (ref. out/open)					0.822	1.216	0.742	1.348	0.725	1.379	0.721	1.388
Personal comfortability with being SGM					0.820	1.219	0.763	1.311	0.721	1.387	0.717	1.394
**Family—Resilience and Discrimination**												
Family cohesion							0.696	1.437	0.544	1.837	0.544	1.838
Family of origin discrimination distress							0.370	2.704	0.291	3.432	0.291	3.434
Parenting discrimination distress							0.460	2.172	0.342	2.923	0.340	2.937
**Community—Resilience and Discrimination**												
Social resources									0.437	2.290	0.436	2.291
Harassment and discrimination distress									0.210	4.769	0.210	4.771
Gender expression discrimination distress									0.182	5.486	0.181	5.513
Victimization discrimination distress									0.292	3.426	0.292	3.429
Vicarious discrimination distress									0.610	1.640	0.609	1.642
Isolation discrimination distress									0.399	2.507	0.399	2.509
**State—Laws and Policies**												
SGM state law score											0.535	1.869
TGSB proposed or enacted (ref. no)											0.553	1.810

VIF = variance inflation factor.

## Appendix B

**Table A2:** 95% confidence intervals for beta coefficients.

Variables	Model 1		Model 2		Model 3		Model 4		Model 5		Model 6	
**Individual—Socioeconomic**	**95% CI**		**95% CI**		**95% CI**		**95% CI**		**95% CI**		**95% CI**	
Constant	21.195	29.984	6.601	14.994	10.402	22.262	11.084	22.328	12.521	23.519	−0.111	0.227
Age	−0.233	−0.119	−0.109	−0.008	−0.064	0.032	−0.055	0.035	−0.061	0.025	12.888	23.920
Hispanic, Latino, or Spanish (ref. non-Hispanic)	−1.165	2.803	−0.376	3.026	−1.222	1.944	−1.903	1.071	−1.761	1.094	−0.062	0.024
Some college, Associate’s degree	−4.270	−0.985	−3.903	−1.087	−3.642	−1.030	−3.268	−0.820	−2.978	−0.621	−1.900	0.959
College graduate 4 years+ (ref. high school grad or less)	−3.436	0.353	−1.570	1.695	−2.059	1.010	−1.332	1.547	−1.638	1.139	−3.009	−0.657
Unemployed	−2.461	2.336	−3.397	0.723	−2.776	1.045	−2.159	1.416	−2.192	1.257	−1.640	1.134
Out of labor force	−1.993	1.299	−1.310	1.512	−1.027	1.589	−0.426	2.025	−0.218	2.138	−2.265	1.182
Unable to work (ref. employed)	−0.475	4.218	−2.395	1.679	−1.175	2.636	−1.062	2.503	−0.765	2.668	−0.168	2.186
White	−2.435	3.721	−1.675	3.600	−2.140	2.753	−1.924	2.651	−2.215	2.191	−0.827	2.609
Black	0.345	7.325	1.636	7.626	0.416	5.975	0.433	5.640	−0.336	4.677	−2.209	2.188
Other (ref. multiple)	−1.394	6.307	−0.226	6.377	−0.403	5.723	−0.720	5.012	−1.312	4.208	−0.326	4.677
$20,000–49,999	−2.402	1.044	−2.508	0.458	−2.462	0.284	−2.430	0.141	−2.360	0.109	−1.238	4.286
$50,000+ (ref. < $20,000)	−2.686	1.248	−1.375	2.008	−1.432	1.700	−1.404	1.531	−1.281	1.546	−2.366	0.099
Other, multiple sexual orientation	−1.790	3.720	−1.835	2.898	−2.140	2.251	−2.469	1.639	−2.636	1.320	−1.219	1.606
Bisexual	−1.126	3.448	−2.494	1.448	−2.331	1.352	−2.715	0.735	−2.728	0.592	−2.596	1.354
Lesbian (ref. gay)	−2.076	3.481	−2.382	2.383	−3.054	1.363	−3.207	0.924	−2.986	0.982	−2.667	0.649
Female	−4.758	−0.482	−4.497	−0.825	−2.867	0.576	−2.336	0.894	−1.620	1.500	−2.935	1.026
Transgender, nonbinary	−2.579	3.263	−4.151	0.889	−3.581	1.087	−2.698	1.677	−2.180	2.132	−1.701	1.417
Other, multiple gender identity (ref. male)	−5.046	2.440	−5.837	0.585	−4.580	1.392	−3.281	2.318	−2.488	3.009	−2.169	2.135
Married	−2.279	1.923	−1.563	2.040	−0.905	2.442	−1.310	1.836	−1.692	1.339	−2.577	2.917
Divorced, widowed, separated (ref. single)	0.497	4.720	−0.394	3.236	−1.260	2.110	−1.603	1.572	−1.460	1.595	−1.732	1.302
**Individual—Mental Health**												
Depression			0.243	0.556	0.166	0.465	0.143	0.422	0.089	0.362	0.091	0.364
Stress			−0.056	0.191	−0.141	0.106	−0.111	0.119	−0.099	0.122	−0.103	0.118
Anxiety			0.343	0.654	0.243	0.536	0.178	0.454	0.158	0.425	0.157	0.423
**Individual—Resilience, Vigilance, and Outness**												
Perception of self					−0.228	−0.001	−0.208	0.004	−0.224	−0.020	−0.229	−0.025
Planned future					−0.250	0.017	−0.251	0.002	−0.183	0.064	−0.181	0.066
Structured style					−0.192	0.045	−0.216	0.007	−0.201	0.013	−0.198	0.016
Social competence					−0.064	0.098	−0.058	0.097	−0.020	0.133	−0.021	0.132
Vigilance discrimination distress					1.096	1.618	−0.368	0.325	−0.480	0.220	−0.461	0.238
Outness (ref. out/open)					1.902	4.247	−0.229	2.102	−0.327	1.921	−0.392	1.855
Personal comfortability with being SGM					−0.303	−0.010	−0.255	0.023	−0.248	0.027	−0.235	0.040
**Family—Resilience and Discrimination**												
Family cohesion							−0.112	0.026	−0.075	0.075	−0.077	0.073
Family of origin discrimination distress							0.302	0.534	0.062	0.343	0.059	0.339
Parenting discrimination distress							0.664	1.355	0.014	0.757	0.018	0.761
**Community—Resilience and Discrimination**												
Social resources									−0.207	−0.060	−0.205	−0.058
Harassment and discrimination distress									−0.295	0.030	−0.298	0.027
Gender expression discrimination distress									−0.005	0.357	0.001	0.363
Victimization discrimination distress									0.560	1.013	0.556	1.008
Vicarious discrimination distress									−0.084	0.070	−0.087	0.067
Isolation discrimination distress									−0.207	−0.060	−0.120	0.218
**State—Laws and Policies**												
SGM state law score											−0.079	−0.003
TGSB proposed or enacted (ref. no)											0.150	2.785

CI = confidence interval.
